# RDS-NExT workshop: consensus statements for the use of surfactant in preterm neonates with RDS

**DOI:** 10.1038/s41372-023-01690-9

**Published:** 2023-05-15

**Authors:** Vineet Bhandari, Rachel Black, Bheru Gandhi, Susan Hogue, Venkatakrishna Kakkilaya, Michel Mikhael, Fernando Moya, Chad Pezzano, Pam Read, Kari D. Roberts, Rita M. Ryan, Richard H. Stanford, Clyde J. Wright

**Affiliations:** 1 The Children’s Regional Hospital at Cooper/Cooper Medical School of Rowan University, Camden, NJ USA; 2 AESARA Inc., Chapel Hill, NC USA; 3 Texas Children’s Hospital, Baylor College of Medicine, Houston, TX USA; 4 Division of Neonatal-Perinatal Medicine, Department of Pediatrics, University of Texas Southwestern Medical Center, Dallas, TX USA; 5 Children’s Hospital of Orange County, Orange, CA USA; 6 Division of Wilmington Pediatric Subspecialists, Department of Pediatrics, UNC School of Medicine, Wilmington, NC USA; 7 Department of Cardio-Respiratory Services Pediatric –Albany Medical Center, Albany, NY USA; 8 University of Minnesota, Minneapolis, MN USA; 9 UH Rainbow Babies and Children’s Hospital –Case Western Reserve University, Cleveland, OH USA; 10 Section of Neonatology, Department of Pediatrics, Children’s Hospital Colorado and the University of Colorado School of Medicine, Aurora, CO USA

**Keywords:** Paediatrics, Respiratory tract diseases

## Abstract

**Objective:**

To provide the best clinical practice guidance for surfactant use in preterm neonates with respiratory distress syndrome (RDS). The RDS-Neonatal Expert Taskforce (RDS-NExT) initiative was intended to add to existing evidence and clinical guidelines, where evidence is lacking, with input from an expert panel.

**Study design:**

An expert panel of healthcare providers specializing in neonatal intensive care was convened and administered a survey questionnaire, followed by 3 virtual workshops. A modified Delphi method was used to obtain consensus around topics in surfactant use in neonatal RDS.

**Result:**

Statements focused on establishing RDS diagnosis and indicators for surfactant administration, surfactant administration methods and techniques, and other considerations. After discussion and voting, consensus was achieved on 20 statements.

**Conclusion:**

These consensus statements provide practical guidance for surfactant administration in preterm neonates with RDS, with a goal to contribute to improving the care of neonates and providing a stimulus for further investigation to bridge existing knowledge gaps.

## Introduction

Respiratory distress syndrome (RDS) in neonates is often defined as respiratory distress occurring within 6 h of birth and associated with radiographic features of a reticular-granular pattern, low lung volumes, and air bronchograms and is caused by an inadequate amount of surfactant [1]. Other definitions use clinical signs and symptoms rather than radiographic data to confirm diagnosis. Although treating neonatal RDS with exogenous surfactant is lifesaving, the variability in criteria used to make the diagnosis contributes to the lack of consensus on treatment thresholds. Surfactant replacement therapy has been demonstrated to improve clinical outcomes, including reductions in mortality risk and risk of air leaks, in neonates with RDS [2, 3].

Guidelines in the US published by the American Association for Respiratory Care (AARC) and the American Academy of Pediatrics (AAP) were last updated almost 10 years ago, in 2013 and 2014, respectively [4, 5]. More recent international guidelines have been published, including the European Consensus Guidelines on the Management of RDS: 2022 Update, the Turkish Neonatal Society Guidelines in 2019, and the Canadian Paediatric Society Position Statement Guideline in 2021 [6–8]. In addition, many neonatal intensive care units (NICUs) create and adhere to their own protocols at the institutional level, for example, the Royal Children’s Hospital, Starship Children’s Hospital, and Alaska Native Medical Center [9–11]. Recent reviews summarize current research and complement existing guidelines [12–14].

Variability among these guidelines demonstrates a lack of consensus concerning surfactant use and administration in the neonatal setting. Variation exists at the institutional level in the management of neonates with RDS, including neonates born at increasingly lower gestational ages, mode of administration (as new, less-invasive, methods are being developed), timing of administration, repeat dosing, and infant positioning during administration of surfactant. Gaps in evidence and shortcomings of existing randomized clinical trials (RCTs) further complicate bedside decision-making, leaving clinicians on their own to answer important questions about the patients for whom they care in the NICU.

The objective of the Respiratory Distress Syndrome Neonatal Expert Taskforce (RDS-NExT) initiative was to define the most-effective practice strategies pertaining to neonatal surfactant use and administration, specifically in preterm neonates with RDS. We approached this by convening a panel of leading experts practicing in varying acuity level NICUs from various regions of the US, in an effort to establish consensus on best clinical surfactant practice. This was accomplished by utilizing a modified Delphi method [15]. The output of this initiative was not intended to replace existing evidence and clinical guidelines; rather, the goal was to fill in the gaps where evidence is lacking, with input from expert opinion leaders, in order to improve the care of neonates with RDS or at high-risk of developing RDS.

## Methods

Independent external consultants (RB, SH, RS, and PR) worked with the committee chair (VB), to generate a list of 33 health care providers (HCPs), including physicians/neonatologists (MDs), nurse practitioners (NPs), and registered respiratory therapists (RRTs), who specialize in neonatal care and administer surfactant routinely in their NICU from published literature, known key opinion leaders, and Delphi method facilitators. The expert panel was selected from this list to include providers from academic, university-affiliated practices, as well as private practice NICUs from various regions in the US.

A targeted review of literature concerning surfactant administration in neonates with RDS was conducted. This was used to inform the development of a web-based survey led by the committee chair (VB) in collaboration with independent external consultants with expertise in Delphi methodology (RB, SH, RS, and PR). The survey consisted of 29 open-ended and 9 multiple-choice questions (Supplementary Table A) and was distributed to panelists using the Survey Monkey platform in advance of the first workshop. Results from the survey were used to develop initial proposed statements around areas of interest that lacked consensus. Statements were divided into 3 sections: Section 1: establishing RDS diagnosis and indicators for surfactant administration; Section 2: surfactant administration methods and techniques; and Section 3: other considerations.

**Fig. 1:**
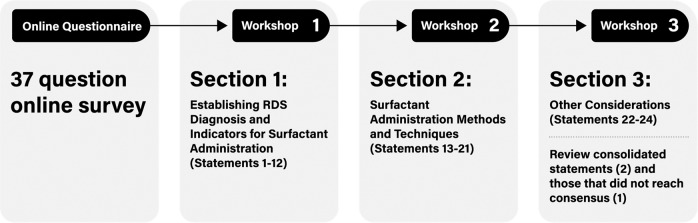
An overview of the modified Delphi process. The figure illustrates the sequence of the initial questionnaire survey, followed by 3 workshops that covered the 3 sections of the RDS guidelines.

**Fig. 2:**
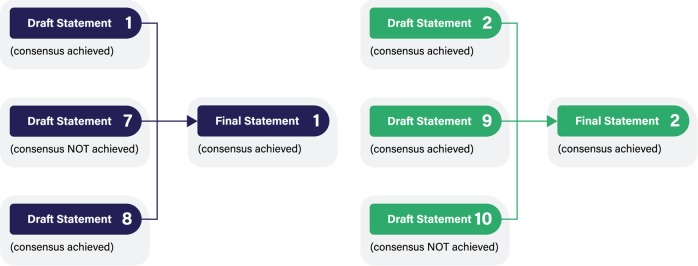
An overview of the consolidation of draft statements into final statements*. The figure illustrates the process of achieving consensus for the specific statements. *Draft Statements 1, 7, and 8 were consolidated into Final Statement 1. Draft Statements 2, 9, and 10 were consolidated into Final Statement 2.

A modified Delphi method was used to obtain expert consensus over the course of 3 live, virtual workshops, during which potential consensus statements were deliberated (Fig. 1). Deliberations sometimes continued thereafter as well. During the workshops, proposed statements were reviewed and discussed. Each initial statement was presented to the group, with relevant data provided by panelists in response to the survey questionnaire. Statements were discussed and modified until panelists moved to vote, selecting their choice on a 5-point Likert scale (strongly disagree, disagree, neutral, agree, or strongly agree). Panelists anonymously submitted their vote for each statement, which was recorded. Consensus was defined a priori as 80% of panelists selecting agree or strongly agree. Section 1 statements were voted on in workshop 1 (April 7, 2022). Section 2 statements were voted on in workshop 2 (April 12, 2022). Section 3 statements were voted on in workshop 3 (May 24, 2022), along with redundant statements from workshops 1 and 2 that were consolidated and statements that did not reach consensus (Fig. 2). In advance of workshop 3, proposed statements were sent out to panelists and feedback was obtained ahead of the meeting. This feedback was incorporated into the proposed statements in advance of workshop 3.

After completion of the 3 workshops, consensus statements and voting results were compiled, and after the receipt of absentee votes, sent out to panelists. During the course of the workshops, panelists discussed the need for additional detailed information regarding the indications for surfactant use. A subcommittee of volunteers from among the workshop panelists was convened to describe parameters and clinical scenarios for which surfactant is indicated for preterm infants with RDS.

## Results

Of the 15 HCPs contacted, 10 agreed to serve on the expert panel. Of these, 9 panelists completed the survey. All 11 panelists (10 plus the committee chair) participated in workshops 1 and 2. Two panelists were unable to attend workshop 3 due to scheduling conflicts, so absentee votes for these panelists were obtained via email. Names of participating panelists can be found in Supplementary Table B.

A total of 24 statements was discussed, refined, and voted on over the 3 workshops. Consensus was achieved on 20 statements. The original statements presented to the group, as well as the final statement and voting results, can be found in Supplementary Table C. The final consensus statements are listed below. They are followed by the panelists’ reasons for agreeing or disagreeing with the statements, as well as their limitations and applicability to clinical practice.

### Section 1: Establishing RDS diagnosis and indicators for surfactant administration

**Statement 1** (consolidated from original statements 1, 7, and 8; see Supplementary Table C): When the clinical decision has been made to administer surfactant, preterm infants with RDS should receive surfactant early (≤2 h of life), preferably within 1 h of life.

A review by Bahadue et al. concluded that early selective surfactant administration within the first 2 h of life (throughout this manuscript, *life* is used to denote *extrauterine life*) in neonates with RDS requiring assisted ventilation decreased the risk of mortality, acute pulmonary injury, and chronic lung disease compared to delayed treatment [16]. Some panelists expressed uncertainty about the 2 h limit. Variability in timing of treatment with surfactant replacement therapy and disease severity of patients included in clinical trials have left clinicians with uncertainty regarding the optimal timing of surfactant treatment [16]. Furthermore, a review that included more-recent trials demonstrating current clinical practice concluded that prophylactic use of surfactant, when compared to routine stabilization using continuous positive airway pressure (CPAP), does not improve clinical outcomes and may increase the risk of chronic lung disease or death [17].

Panelists also discussed the applicability of this statement to various patient populations, including neonates of ≥30 weeks’ gestational age (GA), extremely preterm infants (<28 weeks’ GA) and low birth weight neonates requiring CPAP.

**Statement 2** (consolidated from original statements 2, 9, and 10; see Supplementary Table C): For preterm infants with RDS receiving positive pressure support, an elevated and increasing fraction of inspired oxygen (FiO_2_) is an important indicator of the need for surfactant treatment based on administration criteria; other clinical measures of respiratory distress and/or support, alone or in combination, may precede and preempt FiO_2_ as an indication of need for surfactant treatment.

There was a significant amount of discussion regarding identifying a specific FiO_2_ threshold for surfactant administration, as the evidence is inconsistent. The 2022 European Consensus Guidelines recommendations state that a FiO_2_ of >0.3 be used as a cutoff for surfactant administration for all babies with a clinical diagnosis of RDS, especially in the early phase of worsening disease [6]. Other studies have also utilized a FiO_2_ threshold of 0.3 [18, 19]; however, the populations included in studies supporting this cutoff are not generalizable to the entire population of neonates with RDS. The 0.3 FiO_2_ criterion was derived from observational studies (of CPAP failure, etc.) [18–21]. The need for a specific FiO_2_ threshold spurred much discussion among the group and resulted in the development of the surfactant indicator table (Table 1). Additional considerations included the type of support the infant is on, the surfactant delivery technique (invasive vs less invasive), clinical parameters, and differences in resources available, as per NICU level of care.

**Table 1:** Surfactant indicator table. Considerations for Surfactant Administration for RDS^a^ (Need to fulfill at least 3 of 4 criteria).

Gestational age	23 0/7–27 6/7 Weeks	28 0/7–36 6/7 Weeks
Clinical status (Downes’ Score^b^)	4–7	4–7
Respiratory support status	Intubated at any time^c^ OR NIPPV: MAP ≥ 6; CPAP ≥ 6	Intubated at any time^c^ OR NIPPV: MAP ≥ 7; CPAP ≥ 7
FiO_2_ requirement^d,e^	≥0.3 if early (≤2 h of life) OR ≥0.4 if late (>2 h of life)	≥0.3 if early (≤2 h of life) OR ≥0.4 if late (>2 h of life)
Blood gas status^f^	pH <7.25, pCO_2_ > 60	pH <7.25, pCO_2_ > 60

EARLY (≤2 h of life); LATE (>2 h of life).

*CPAP* continuous positive airway pressure, *ETT* endotracheal tube, *INSURE* INtubation-SURfactant-Extubation, *FiO*_*2*_ fraction of inspired oxygen, *LISA* less-invasive surfactant administration, *LMA* laryngeal mask airway, *MAP* mean airway pressure, *MIST* minimally invasive surfactant administration, *NIPPV* nasal intermittent positive pressure ventilation, *pCO*_*2*_ partial pressure of carbon dioxide, *RDS* respiratory distress syndrome, *SpO*_*2*_ oxygen saturations (pulse oximetry).

^a^History, signs, and symptoms consistent with surfactant deficiency.

^b^Details of the Downes’ scoring system is shown in Supplementary Table D. Briefly, includes 0-2 points each for Respiratory Rate (60, 60–80, 80), Retractions (0, mild, severe), Cyanosis (no in room air, relieved by O_2_, yes even with O_2_), Air Entry (good, mildly decreased, none), Grunting (non, w/stethoscope, audible).

^c^If infant is intubated for resuscitation or meets other clinical parameters for surfactant administration, consider giving surfactant before extubating.

^d^To maintain a preductal SpO_2_ of 90–95%. Infants transitioning to extrauterine life and stable on CPAP in the delivery room may require a higher FiO_2_ temporarily that may not necessarily warrant surfactant administration. Severe respiratory distress, even with a lower FiO_2_, may warrant earlier surfactant administration.

^e^Evaluate the risk of the intervention for surfactant administration (via LISA-MIST/INSURE/LMA vs ETT), followed by the risk of exposure to invasive mechanical ventilation.

^f^Arterial or capillary blood gas, only if available.

The type of support the infant is on (e.g., CPAP vs mechanical ventilation) should be considered. The use of a lower threshold negates the work from the COIN and SUPPORT trials for noninvasive ventilation (NIV) alone, as these studies suggest that a substantial number of babies can be supported on CPAP and a higher level of oxygen [22, 23]. It must be recognized that a lower FiO_2_ criterion results in earlier treatment in babies with true surfactant deficiency; however, it also increases exposure to risks associated with surfactant administration in the subset of babies who would have never met treatment threshold if the FiO_2_ criteria were set higher.

This balance of risk vs benefit is also necessarily dependent on the risk profile associated with the different modes of surfactant administration. When administered via an endotracheal tube (ETT) followed by invasive mechanical ventilation (IMV), a higher FiO_2_ threshold may be more appropriate. A lower threshold may be more appropriate when an infant is on NIV and surfactant is administered and not followed by IMV, such as with less-invasive surfactant administration (LISA), INtubation-SURfactant-Extubation (INSURE), minimally invasive surfactant administration (MIST), or laryngeal mask airway (LMA) surfactant delivery. When the need for surfactant administration comes with a high likelihood of prolonged mechanical ventilation if an ETT is placed or a practitioner is not comfortable with the use of LISA and/or LMA to deliver surfactant, then a higher threshold of FiO_2_ may be preferred [14, 24].

Clinical parameters should also be considered. However, work of breathing (WOB) is very subjective and has not been studied alone.

Differences in access to resources as per NICU level of care and how elevation (altitude) of the NICU site impacts FiO_2_ requirement need to be considered as well.

**Statement 3**. Chest x-ray (CXR) confirmation for RDS diagnosis is suggested but not required prior to surfactant administration.

CXRs can be useful to rule out pneumothorax; however, a CXR may not be available during transport or when resources are limited (e.g., night shift) and could cause delays in surfactant administration. Recent approaches with no radiation exposure, such as lung ultrasound, do not require CXR for confirmation of the diagnosis of RDS [25–31].

**Statement 4**. The most important predictors for determining the need for surfactant in preterm infants with RDS are GA, FiO_2_ requirement and clinical signs and symptoms with supporting investigations (e.g., CXR, blood gases).

The biggest predictor for RDS is GA [32–34]; however, the biggest predictor for noninvasive ventilation failure is FiO_2_ [18–20].

**Statement 5**. A second or third dose of surfactant may be necessary for ongoing RDS depending on clinical factors (e.g., lack of improvement, FiO_2_ requirement, increased WOB, or continued need for mechanical ventilation).

Limited data exist around indications or outcomes for repeat doses [35–37]. Findings from a review by Soll et al. suggest that infants with ongoing respiratory insufficiency who received multiple doses of surfactant had better clinical outcomes than those who received single dosing [38]. Repeat doses, in accordance with manufacturer’s guidelines, may be necessary in some situations and should not be discouraged or delayed, if clinically indicated.

**Statement 6**. Based on current data, GA alone should not be the sole criterion for surfactant administration.

The decision to administer surfactant should not be based on GA alone. No data exist for dosing solely based on GA; however, the younger the GA of the infant, the higher the risk for RDS and noninvasive respiratory support failure [39]. RCTs comparing initial stabilization on CPAP vs intubation in babies born at <28 weeks’ GA demonstrate that a significant majority (~50–70%) of those managed on CPAP will receive surfactant [22, 40]. Results from a German study are comparable [41].

As discussed in **Statement 2**, the risk-benefit profile of the mode of administration should be considered. Specifically, surfactant administered via ETT, followed by exposure to IMV potentially carries a higher risk profile than administration via LISA, MIST, INSURE, or LMA [42]. Meta-analyses suggest that LISA is superior to the INSURE technique in terms of avoidance of BPD and IVH [41]. Furthermore, the GA of the baby independently modifies the risk profile associated with mode of administration. For example, chronic lung disease affects lower GA infants at higher rates [43]. Given the relationship between IMV and chronic lung disease [44], the risk of adverse outcomes increases among lower GA infants administered surfactant via ETT followed by IMV. A recently published article showed that LISA in the delivery room is routine practice in Germany for infants <27 weeks’ GA [45]. There is an ongoing single-center study to evaluate delivery room LISA vs NICU LISA [46].

Additionally, extremely early GA should be considered, as infants at 22–23 weeks’ gestation have almost universally been shown to fail CPAP [47].

**Statement 7** (originally 11; see Supplementary Table C): Additional studies are needed to assess the role of lung ultrasound and clinical respiratory scoring as adjunct tools in determining the need for surfactant administration for non-intubated infants with RDS.

Although more popular in Europe, use of lung ultrasound is becoming more commonplace in the US [48–50]. An early high lung ultrasound score correlates with the need for surfactant administration in preterm neonates with RDS [51]. Lung ultrasound score has also been correlated with oxygenation status and may help predict bronchopulmonary dysplasia (BPD) [52].

Many methods to assess the severity of respiratory distress exist, including Downes’ score [53], Silverman Anderson [54], and the Respiratory Severity Score; there is no consensus on which is the ideal scoring system [55–57]. Use of these tools should not delay therapy.

**Statement 8** (originally 12; see Supplementary Table C): Barriers to the timely administration of surfactant may be due to limited availability of appropriately skilled staff and/or resources (e.g., delay in diagnosing RDS and timely transport to a regional center).

Barriers to timely surfactant administration include limited availability of appropriately skilled staff (ability to intubate, familiarity with less-invasive methods of surfactant administration) and/or resources (equipment and surfactant medication), as well as delays in diagnosing RDS (need for confirmatory radiological exams, transport to a regional center) [58].

These barriers may not be an issue for NICUs providing level III or higher care; however, it is unknown how often surfactant is delayed in other settings, as data do not exist.

### Section 2: Surfactant administration methods and techniques

**Statement 9** (originally 13; see Supplementary Table C): Surfactant can be administered using equipment based on the provider’s experience/skill level, preference, and institutional practice.

The type of equipment used for surfactant administration may influence procedure duration, number of attempts, accuracy of dose delivery, and need to interrupt the delivery of positive pressure during the procedure; however, no RCTs have been performed to determine the optimal delivery method. Designing such a trial would be difficult and would require consideration of the environment, the skill level of the operator, and the target patient population. Kribs et al. report findings from a non-blinded study that compared outcomes among infants who received surfactant via a thin endotracheal catheter during CPAP-assisted spontaneous breathing (LISA; intervention group) vs those who received surfactant after conventional endotracheal intubation during mechanical ventilation (control group) [59]. The primary outcome, survival without BPD, was not demonstrated; however, some of the secondary endpoints (rates of successful application and number of attempts, duration of mechanical ventilation, etc.) demonstrated important safety benefits associated with LISA administration. Infants who received surfactant via the LISA method were less frequently intubated (80 infants [74.8%] vs 103 [99.0%]; *P* < 0.001) and required fewer days of mechanical ventilation compared to those in the control group. The groups had a similar number of surfactant doses per infant and more than 1 attempt for successful surfactant administration was needed in 27% of the infants in both groups [59]. In a consensus guideline for LISA, Reynolds et al. concluded that LISA can be a safe method, with the potential to improve outcomes for premature neonates [60]. In a recent RCT, MIST therapy compared with sham (control) treatment did not significantly reduce the incidence of the primary composite outcome of death or BPD, though the incidence of BPD was significantly lower in the MIST group (*P* = 0.03) [61]. Further, a meta-analysis comparing different surfactant strategies concluded that administration of surfactant via thin catheter, compared with ETT administration, was associated with a reduced risk of BPD/death, fewer patients intubated in the first 72 h, and reduced incidence of morbidities and in-hospital mortality [62]. The above data suggest that treatment with surfactant via thin catheter may be preferable to ETT administration; however, additional studies of adequate size and power are needed to confirm these findings [62]. Furthermore, given subgroup differences observed in some of the RCTs referenced above, whether the protective effect of LISA/MIST varies across GA strata remains to be determined.

**Statement 10** (originally 14; see Supplementary Table C): Routine repositioning during surfactant administration may not improve the distribution of surfactant relative to maintaining the infant supine; repositioning may increase the risk of device malposition.

There are no RCTs comparing response with repositioning of the infant during surfactant administration. Uncertainty exists around distribution of surfactant within the lungs and safety concerns arise with the repositioning of a critically ill infant. In a recent survey of US HCPs, 44% turn or reposition the infant during surfactant administration [63]. Manufacturer guidelines are available as they relate to infant positioning [64–66].

Preclinical data in a lamb model demonstrate that 1 aliquot of exogenous surfactant administered via the ETT distributes evenly without repositioning. There are data for 4 vs 2 doses, with no differences seen [67]; however, similar data in humans do not exist.

**Statement 11** (originally 15; see Supplementary Table C): Surfactant can be administered as a single bolus or divided aliquots, based on provider preference, mode of administration, manufacturer recommendation, and clinical considerations.

Some centers may defer to manufacturer recommendations, which recommend using multiple aliquots [64–66]. There was an RCT comparing 2 aliquots vs 4 aliquots of Survanta®. It showed no major differences [68]; however, the studies were conducted more than 20 years ago and may not be applicable to current NICU populations. In a recent survey of US HCPs, 50% said they use 2 aliquots; 32%, a single bolus dose; 7%, >2 aliquots; and 11% reported “other” [63]. The rationale most often provided for using the single bolus dose was tolerability and maintaining the infant in a neutral position. For providers who use 2 aliquots, historical practice was most frequently noted, with tolerability being second most common rationale. HCPs who reported “other” mentioned type of surfactant used, location, GA/weight, and clinical presentation in determining how to administer surfactant. Bolus administration of surfactant is preferred when given via the ETT; using the LISA approach, surfactant administration can usually to be completed in <1 min, though it might require more time if apnea/bradycardia/surfactant reflux occur [8, 69, 70].

**Statement 12** (originally 16; see Supplementary Table C): When using the INSURE technique to administer surfactant, infants should be extubated as soon as possible.

The AAP guidelines do not give a time recommendation for extubation after surfactant administration via INSURE – just that it should be rapid [5]. The experts agreed that the most likely protective effect gained through administration via INSURE is limited IMV exposure. Some centers do not specify when to extubate after surfactant administration after the INSURE procedure has been completed. If premedications were used with the INSURE technique, such as sedative and/or paralyzing agents, this may influence timing of extubation after surfactant has been administered.

**Statement 13** (originally 17; see Supplementary Table C): For spontaneously breathing infants with RDS on CPAP for whom the decision to give surfactant has been made, less-invasive methods of surfactant administration may be appropriate alternatives to the INSURE technique.

In a network meta-analysis, Isayama et al. reported that the use of LISA was associated with a lower likelihood of the primary outcome of death or BPD than was mechanical ventilation or nasal CPAP alone. INSURE was associated with a lower likelihood of death or BPD than was mechanical ventilation or nasal CPAP alone. Ranking probabilities supported LISA as the best among all strategies for all outcomes assessed. INSURE tied with nasal intermittent positive pressure ventilation as the second-best strategy to prevent death or BPD [71]. However, the authors judged the quality of evidence of the meta-analysis to be low because the sample size of all included studies did not reach the optimal information size [71]. The limitations of the OPTIMIST-A trial comparing MIST vs CPAP and the LMA vs INSURE trials must be acknowledged [61, 72]. A consensus guideline from the UK has suggested that LISA has the potential to “improve outcomes” for preterm infants with RDS [60]. Although promising, these reports are not definitive and may not be applicable in certain GA categories. The consensus of this expert panel was that a single approach does not fit all NICU sites/scenarios. At the current time, the choice of delivery method should be made with careful consideration of the environment, resources available, operator experience, and patient characteristics, including GA and degree of illness. The clinician caring for the infant should choose which method of surfactant administration is most appropriate. It is likely that additional studies will further inform this practice.

**Statement 14** (originally 18; see Supplementary Table C): For preterm infants with RDS with adequate respiratory effort on CPAP (not requiring IMV), LISA/MIST are appropriate less-invasive options for surfactant administration based on provider experience and institutional practice.

There is emerging evidence for LISA/MIST modes of administration of surfactant to be considered preferential [14, 45, 71, 73, 74]. It is important to note that most studies of these modes of surfactant delivery included early treatment at relatively low thresholds for administration (e.g., FiO_2_ of 0.3) and they have not been studied for rescue delivery in unstable neonates.

A meta-analysis of 6 RCTs comparing surfactant administration utilizing LISA vs standard endotracheal administration showed a reduction in the composite outcome of death or BPD, BPD among survivors, and need for mechanical ventilation at 72 h and at any time during the NICU course. There were no differences found in death or other neonatal morbidities. Three of the six studies included patients <28 weeks, with one including 23-week preterm infants [75].

**Statement 15** (originally 19; see Supplementary Table C): Surfactant administration via supraglottic airway devices (e.g., LMA) may benefit certain populations of preterm infants and is a promising method of surfactant administration.

A study by Roberts et al. found that infants of 28 0/7–35 6/7 weeks gestational age and with birth weights ≥1250 g who received surfactant via LMA had a decreased rate of intubation and mechanical ventilation compared with controls (38% vs 64%; OR: 0.30 [95% CI, 0.13–0.70]; *P* = 0.006) [72]. A recent meta-analysis of 6 RCTs found that administering surfactant via LMA was associated with decreased FiO_2_ requirement, decreased intubation, and decreased mechanical ventilation [76]. A recent study comparing LMA to INSURE in infants of 27–36 weeks’ gestation and weighing >800 g found surfactant therapy via LMA was non-inferior to INSURE for efficacy and that LMA administration decreased early failures, possibly by avoiding adverse effects of premedication, laryngoscopy, and intubation [77]. Notably, both groups received atropine premedication while only the INSURE group received remifentanil premedication.

**Statement 16** (originally 20; see Supplementary Table C): More data are required to evaluate the use of the promising technique of aerosol administration of surfactant.

Although aerosolization is the most noninvasive of all methods of surfactant administration, studies have historically been unable to show improvement in respiratory status [78–83]. A recent study was the first to show benefit, with a reduction in intubation and surfactant via endotracheal instillation of nearly one-half (26% in the aerosol group and 50% in the usual care group; *P* < 0.0001) [84]. There is currently no US Food and Drug Administration–approved device available.

**Statement 17** (originally 21; see Supplementary Table C): Device development research and more experience with novel methods of surfactant administration (e.g., LMA, aerosol) is needed for smaller, less-mature infants.

The study by Roberts et al. included only infants of 28 0/7–35 6/7 weeks’ gestation and weighing ≥1250 g [72]. LMAs are currently available only for infants weighing ≥1250 g. Devices appropriate for smaller infants are needed.

### Section 3: Other considerations

**Statement 18** (originally 22; see Supplementary Table C): Premedication usage for surfactant administration depends on the method of administration. Pain management, physical discomfort, procedural success, and minimization of adverse events are all considerations around premedication usage in surfactant administration.

Discrepancies in the use of premedication prior to surfactant administration exist across clinical institutions [63, 85–88]. While early caffeine has been recommended, the exact timing of caffeine initiation has not been specified [89]; more research is needed in this area [90, 91].

**Statement 19** (originally 23; see Supplementary Table C): More studies are needed to evaluate the safety and efficacy of sucrose use as a premedication prior to administration of surfactant.

Oral sucrose solution has been used as a pain medication with LISA/MIST [14] and LMA [72] administration of surfactant.

**Statement 20** (originally 24; see Supplementary Table C): Intravenous (IV) atropine is not routinely used for less-invasive methods of surfactant administration.

IV atropine has been used to minimize bradycardia during nonurgent intubation and/or LISA [92, 93]. Use of atropine may mask prolonged hypoxia. This risk is related to the use of laryngoscopy. Ultimately, surfactant administration should not be delayed just for the purpose of establishing IV access for atropine administration.

## Discussion

Consensus statements for the use of surfactant in infants with RDS were generated utilizing a modified Delphi method. This is a standard methodology to obtain consensus from a group of experts and has been used by neonatal specialists in the UK to generate consensus statements for surfactant replacement therapy in RDS [94]. It has also been used in other therapeutic areas [95–97]. Consensus statements are usually needed when clinical evidence, such as clinical trial data, is lacking regarding treatment decisions, when guidelines may not reflect current clinical practice, or when there are no acceptable standard practice methods. A study by Patel et al. reported the results of interviews with 54 HCPs (neonatologists, NPs, and RRTs) on how they administered surfactant therapy in infants with RDS; the authors concluded that there were no standard practices in respiratory management and surfactant administration [63]. This highlights the need for recommendations such as the consensus statements generated by the RDS-NExT panel to help clinicians better manage RDS when surfactant administration is indicated.

The consensus statements developed by the RDS-NExT panel are not meant to replace guidelines, which are usually developed based on strong clinical evidence, but instead are intended to supplement the guidelines with prevailing clinical practice standards used by experienced clinicians and researchers. Merging clinical practice with available data can help advance neonatal practice and improve patient care.

Given the variability in the criteria (clinical signs and symptoms, with or without radiographic features) used for the diagnosis of RDS, clinicians are left to use proxies to determine the need for surfactant treatment. These include FiO_2_ requirement and level of noninvasive pressure support needed to maintain cardiorespiratory stability. These topics generated vigorous discussion among the panelists.

Establishing a FiO_2_ cutoff value was a subject of much debate. Some panelists felt that using 0.3 as a minimum cutoff value was inappropriate because other important factors that should be considered might lead a clinician to treat with surfactant at a lower FiO_2_. These included the GA of the baby and the subsequent risk of chronic lung disease, the type and level of respiratory support the infant is receiving, the surfactant delivery technique and associated risk profile of administration, clinical parameters (including history of antenatal corticosteroid administration and cardiovascular stability), and the level of NICU care being provided at a particular site. Although a specific value was not included in a consensus statement, the surfactant indicator table (Table 1) includes a range (0.3–0.4), depending on whether the infant is within 2 h of life (early) or more than 2 h (late) after birth. The primary goal is early and appropriate treatment of infants whose respiratory distress is caused by surfactant deficiency. A similar debate was had regarding the appropriate positive pressure cutoff value. Panelists felt that too many other factors needed consideration, for example that use of a higher CPAP would decrease the FiO_2_ requirement. Given these complex clinical relationships, a range (6 vs 7 MAP/CPAP or higher) was ultimately included in the surfactant indicator table (Table 1).

Finally, the panel discussed surfactant administration modes at length. This issue is critically important, and any risk associated with surfactant delivery can blunt its powerful treatment effect. A recent review suggests that minimally invasive methods of surfactant administration have the potential for widespread use in the US because of the associated improved neurodevelopmental outcomes [14]. Evidence on LMA administration is emerging [72, 98]. Aerosolized surfactant is an area of active study [81, 99]. Knowledge gained in these areas through further study will likely refine our use of surfactant in the upcoming years.

The surfactant indicator table was generated with the goal of including a range of clinical presentations, with a level of detail that was not possible to include in the 20 consensus statements. It is the hope of the authors that this table can be used to provide guidance for the use of surfactant in preterm neonates with RDS at individual institutions. Developed in 1970, the Downes’ respiratory scoring system [53] is still in use; however, it has not been standardized for use in extremely preterm neonates. Despite this and consideration of other clinical scoring systems [54], the Downes’ scoring system was selected because it correlates well with blood gas analysis and degree of distress and is used in clinical practices around the world [100–105].

The goal of developing these consensus statements was to consider the general neonatal RDS patient population; therefore, clinical outliers are not discussed. It was not feasible to take into account variations in treatment for the entire spectrum of clinical presentations. It is important to highlight the fact that individual clinical parameters and resources available at a specific site should always be taken into account when considering the indication and mode of surfactant administration. How these factors apply to a particular institution may vary at community and private practices and academic centers. Furthermore, transport availability is a factor that needs to be considered when applying this guidance.

There are some limitations. The initial literature review used to develop the statements for expert review was neither exhaustive nor systematic. In addition, while many important factors regarding the administration of surfactant to neonates with RDS were discussed, other important factors, such as type and dose of surfactant, were not included due to time constraints. Key repetitive themes were identified in the recent literature and these issues were supplemented with unbiased, anonymous input from key opinion leaders, based on their expertise. These comments were collated and provided for all panelists to review. Responses to the survey were anonymous and reviewed by the entire panel, with a trained facilitator allowing all voices to be heard in the 3 live, virtual workshops. Voting at each stage of the virtual workshops was anonymous and was then discussed as a group and edited live by the panelists. This methodology allowed for frank, inclusive input and dialog among all panelists.

Consensus on some statements was difficult to achieve, given the lack of published data and variation in practice methods among the panelists and their home institutions. The panelists considered data from RCTs, review articles, manufacturer recommendations (which are from 2 to 3 decades ago and may not necessarily reflect current practice), and personal experience/preference to develop the final statements.

A key strength was the experienced group of neonatologists plus inclusion of an experienced RRT and NP. These experts, from fairly diverse geographic regions and practice parameters in a wide array of private practice and academic settings, provided information regarding the use of surfactant in preterm neonates with RDS, which we believe provides a degree of generalizability for NICUs in the US.

The goal for these consensus statements was to reach agreement on key topics identified as knowledge and clinical care gaps that have emerged since the last set of guidelines was published in the US nearly a decade ago [4, 5]. It is our hope that these statements provide practical guidance and improve the care of neonates. In addition, this report serves as a stimulus to drive further investigation around existing knowledge gaps.

## Supplementary information

Supplemental Material

## Data Availability

All data generated or analyzed during this study are included in this published article [and its supplementary information files]. Any additional information about the current study are available from the corresponding author on reasonable request.
