# Supplementary material for: RDS-NExT workshop: consensus statements for the use of surfactant in preterm neonates with RDS
Source: J Perinatol. 2023 May 15;43(8):982–90. doi: 10.1038/s41372-023-01690-9 (PMC10400415; doi:10.1038/s41372-023-01690-9)
Supplement: Supplementary file 1 — Supplemental Material [file 41372_2023_1690_MOESM1_ESM.docx]

**Supplement**

1. **Pre-Workshop Questionnaire**

The survey consists of the following four sections:

- Section 1: Baseline Provider Information
- Section 2: Establishing RDS Diagnosis and Indicators for Surfactant Administration
- Section 3: Administration Methods and Techniques
- Section 4: Other Considerations

*Section 1: Baseline Provider Information*

1. Please enter the title of your current role.
2. How many years of clinical practice experience do you have with using surfactants in neonates?
3. What type of institution do you currently work in? (Multiple choice)
   1. Academic medical center
   2. Community hospital
   3. Both

*Section 2: Establishing RDS Diagnosis and Indicators for Surfactant Administration*

1. Is chest x-ray (CXR) confirmation needed for RDS diagnosis prior to surfactant administration for ALL babies in your unit? Please describe why or why not.
2. Does administering surfactant early in the course of RDS improve outcomes? Which outcomes, specifically?
3. How would you define early rescue surfactant administration?
4. Please indicate your level of agreement: Earlier surfactant administration may help to avoid prolonged invasive/non-invasive mechanical ventilation resulting in a common goal to improve outcomes. (Multiple choice)
   1. Strongly disagree
   2. Disagree
   3. Neutral
   4. Agree
   5. Strongly agree
5. What are the three most important indicators (clinical and demographic factors) to determine the use of surfactant?
6. Is FiO_2_ the main indicator for surfactant use? (Multiple choice)
   1. Yes
   2. No
   3. Maybe (Please elaborate)
7. At what threshold FiO_2_ should infants be treated with surfactant?
8. Please indicate your level of agreement: An FiO_2_ exceeding 0.3 within 2 hours of age with adequate CPAP support indicates that surfactant should be administered. (Multiple choice)
   1. Strongly disagree
   2. Disagree
   3. Neutral
   4. Agree
   5. Strongly agree
9. Is there a certain gestational age range below which surfactant should be administered immediately or in the delivery room, irrespective of needing intubation for resuscitation?
10. After the first 2 hours of age, what are the indicators for surfactant administration for RDS?
11. What other meaningful tools/measures are useful for determining surfactant administration (e.g., lung ultrasound, respiratory severity score, etc.)?
12. What are the specific considerations for surfactant administration in subpopulations of neonates (e.g., certain gestational age groups)?
13. From a surfactant administration perspective, how would you classify clinically meaningful gestational age cohorts?
14. What are the barriers to the timely administration of surfactant?
15. Is there anything else related to the timing of surfactant administration that wasn’t mentioned? (If no, leave blank)

*Section 3: Administration Methods and Techniques*

1. Does the type of equipment (MAC, 5-Fr feeding tube) used for surfactant administration matter? Please describe why or why not.
2. Do you routinely reposition the baby during surfactant administration? Please describe why or why not.
3. Do you prefer using a single bolus or divided aliquot dosing when administering surfactant? (Multiple choice)
   1. Single bolus
   2. Divided aliquots
   3. Other (please specify)
4. Follow up: Why do you prefer that method?
5. Do you practice the INSURE (INtubation-SURfactant-Extubation) technique?
6. If you do use INSURE, how soon should babies be extubated?
7. Please indicate your level of agreement: For spontaneously breathing infants on continuous positive airway pressure (CPAP) with RDS, less-invasive methods of surfactant administration are appropriate alternatives for the INSURE technique. (Multiple choice)
   1. Strongly disagree
   2. Disagree
   3. Neutral
   4. Agree
   5. Strongly agree
8. When is less-invasive surfactant administration (LISA) using a vascular or specially-designed catheter/minimally invasive surfactant therapy (MIST) using a feeding tube an appropriate option for surfactant administration?
9. When is laryngeal mask airway (LMA) an appropriate option for surfactant administration (e.g., babies over a certain weight)?
10. When is aerosol administration of surfactant appropriate?
11. What are current knowledge gaps for the above alternative surfactant administration methods (LMA and aerosol)?

*Section 4: Other Considerations*

1. Please indicate your level of agreement: Criteria for redosing surfactant are similar to criteria for initial dose. (Multiple choice)
   1. Strongly disagree
   2. Disagree
   3. Neutral
   4. Agree
   5. Strongly agree
2. Do you routinely premedicate before using the following administration methods: INSURE, LISA/MIST, LMA, other (please specify).
3. What factors impact your decision to use or not use premedication for each selected method?
4. Is sucrose routinely administered before administration of surfactant? Why or why not?
5. If yes, for which administration methods do you use sucrose as premedication? (Conventional mechanical ventilation, INSURE, LISA/MIST, LMA, other [please specify])
6. Is IV atropine routinely administered before administration of surfactant? Why or why not?
7. For which administration methods do you use IV atropine as premedication? (Select all that apply)

a. Conventional mechanical ventilation

b. INSURE

c. LISA/MIST

d. LMA

e. Other (please specify)

1. Do you have any other comments or anything else to add? (If no, leave blank).
2. **List of Panelists**

| **Name** | **Position** | **Geographic Location** | **Role** |
| --- | --- | --- | --- |
| Vineet Bhandari, MD, DM | Division Head, Neonatology, The Children’s Regional Hospital at Cooper; Professor of Pediatrics, Obstetrics, Gynecology and Biomedical Sciences, Cooper Medical School of Rowan University | Camden, NJ | Chair |
| Bheru Gandhi, MD | Assistant Professor of Pediatrics, Texas Children’s Hospital, Baylor College of Medicine | Houston, TX | Panelist |
| Fernando Moya, MD | Director, Division of Wilmington Pediatric Subspecialists, Department of Pediatrics, UNC School of Medicine | Wilmington, NC | Panelist |
| Kari Roberts, MD | Neonatologist, Associate Professor of Pediatrics, University of Minnesota | Minneapolis, MN | Panelist |
| Venkatakrishna Kakkilaya, MD | Attending Physician, Neonatal-Perinatal Medicine at Parkland Memorial Hospital; Associate Professor of Pediatrics at UT Southwestern Medical Center | Dallas, TX | Panelist |
| Clyde Wright, MD | Associate Professor of Pediatrics, Section of Neonatology at University of Colorado School of Medicine | Aurora, CO | Panelist |
| Rita Ryan, MD | Professor of Pediatrics (Neonatology), UH Rainbow Babies and Children’s Hospital – Case Western Reserve University | Cleveland, OH | Panelist |
| Michel Mikhael, MD, FAAP | Medical Director, Children’s Hospital of Orange County Small Baby Unit; Assistant Professor, UC Irvine | Orange, CA | Panelist |
| Jill Herr, MSN NNP-BC | Neonatal Nurse Practitioner, Connecticut Children’s Medical Center | Hartford, CT | Panelist |
| Munish Gupta, MD | Neonatologist, Beth Israel Deaconess Medical Center; Assistant Professor, Harvard Medical School | Boston, MA | Panelist |
| Chad Pezzano, MA, RRT-NPS | Department of Cardio-Respiratory Services Pediatric/Neonatal Manager, Albany Medical Center | Albany, NY | Panelist |

1. **Initial statements, final statements and voting results**

| **Proposed Statement** | **Final Statement** | **Result** |
| --- | --- | --- |
| **Section 1 (Workshop 1A)** | | |
| 1. Administration of surfactant early in preterm infants with RDS improves outcomes. | 1. In preterm infants with a diagnosis of RDS, when the clinical decision has been made to administer surfactant, giving surfactant early (≤2 hours of life) improves outcomes. | Consensus achieved (0 strongly disagree, 1 disagree, 0 neutral, 4 agree, 6 strongly agree) |
| 7. Early surfactant therapy (administered within 2 hours of life) is appropriate for any infant with RDS who meets the dosing criteria. | 7. Early surfactant therapy (administered within 2 hours of life) is appropriate for any preterm infant with RDS who meets the dosing criteria. | **Consensus NOT achieved** (1 strongly disagree, 2 disagree, 0 neutral, 8 agree, 0 strongly agree) |
| 8. All preterm infants intubated for respiratory support due to RDS should be treated with surfactant therapy based on dosing criteria. | 8. All preterm infants intubated due to RDS should be treated with surfactant therapy based on dosing criteria. | Consensus achieved (0 strongly disagree, 0 disagree, 0 neutral, 5 agree, 6 strongly agree) |
| **Note: statements 1, 7, and 8 were combined into statement 178 and voted on during workshop 2* | | |
| 2. For preterm infants with RDS, an elevated FiO2 (e.g., generally ≥ 0.3) is an important indicator for the need of surfactant treatment; other clinical measures of respiratory distress and/or support, alone or in combination, may precede and preempt FiO2 as indication of need for surfactant treatment. | 2. For preterm infants with RDS, an elevated FiO2 while on respiratory support is an important indicator for the need of surfactant treatment; other clinical measures of respiratory distress and/or support, alone or in combination, may precede and preempt FiO2 as indication of need for surfactant treatment. | Consensus achieved (0 strongly disagree, 0 disagree, 0 neutral, 6 agree, 5 strongly agree) |
| 9. After the first 2 hours of life, work of breathing and FiO2 ≥ 0.3 are strong indicators for surfactant treatment for RDS based on dosing criteria. | 9. After the first 2 hours of life, increased work of breathing on positive pressure support, and/or elevated FiO2 are important indicators for surfactant treatment for RDS based on dosing criteria. | Consensus achieved (0 strongly disagree, 2 disagree, 0 neutral, 5 agree, 4 strongly agree) |
| 10. Surfactant should be administered within 2 hours of life in preterm infants with RDS who have FiO2 ≥ 0.3 with adequate respiratory support. | 10. Surfactant should be administered within 2 hours of life in preterm infants with RDS who require FiO2 ≥ 0.3 with adequate positive pressure respiratory support meeting dosing criteria. | **Consensus NOT achieved** (3 strongly disagree, 0 disagree, 2 neutral, 4 agree, 2 strongly agree) |
| **Note: statements 2, 9, and 10 were combined into statement 2910 and voted on during workshop 2* | | |
| 3. Chest x-ray (CXR) confirmation for RDS diagnosis is recommended but not required prior to surfactant administration. | 3. Chest x-ray (CXR) confirmation for RDS diagnosis is suggested but not required prior to surfactant administration. | Consensus achieved (0 strongly disagree, 0 disagree, 1 neutral, 4 agree, 6 strongly agree) |
| 4. The three most important indicators when determining the use of surfactant for preterm infants with RDS are 1. gestational age 2. FiO2 requirement & 3. clinical signs and symptoms (e.g., work of breathing, CXR). | 4. The most important indicators when determining the use of surfactant for preterm infants with RDS are gestational age, FiO2 requirement & clinical signs and symptoms (eg, work of breathing, CXR). | Consensus achieved (1 strongly disagree, 0 disagree, 1 neutral, 4 agree, 5 strongly agree) |
| 5. A second or third dose of surfactant may be necessary for ongoing RDS dependent on clinical factors (e.g., lack of improvement, FiO2, increased work of breathing, or continued need for mechanical ventilation) | 5. A second or third dose of surfactant may be necessary for ongoing RDS dependent on clinical factors (eg, lack of improvement, FiO2 requirement, increased work of breathing, or continued need for mechanical ventilation) | Consensus achieved (0 strongly disagree, 0 disagree, 0 neutral, 5 agree, 6 strongly agree) |
| 6. Gestational age alone is not the sole criterion for immediate surfactant administration. | 6. Based on current data, gestational age alone should not be the sole criterion for surfactant administration. | Consensus achieved (0 strongly disagree, 0 disagree, 0 neutral, 2 agree, 9 strongly agree) |
| 11. Lung ultrasound and respiratory severity score (RSS) may play a role in determining which patients are candidates for surfactant administration. | 11. Lung ultrasound and clinical respiratory scoring (e.g., RSS) may play a role in determining which patients are candidates for surfactant administration. | **Consensus NOT achieved** (0 strongly disagree, 2 disagree, 2 neutral, 5 agree, 2 strongly agree) |
| 12. Overcoming barriers such as limited nearby availability of appropriately skilled staff and resources, including equipment and surfactant medication and/or confirmatory radiological exams may increase the timely administration of surfactant. | 12. Barriers to the timely administration of surfactant may be due to limited availability of appropriately skilled staff and resources (eg, delay in diagnosing RDS and timely transport to a regional center). | Consensus achieved (0 strongly disagree, 1 disagree, 1 neutral, 7 agree, 2 strongly agree) |
| **Section 2 (Workshop 1B)** | | |
| 13. Surfactant can be administered using equipment based on the provider’s experience/preference and institutional practice. Type of equipment may influence duration, number of attempts, accuracy of delivering the dose and need to discontinue positive pressure. | 13. Surfactant can be administered using equipment based on the provider’s experience/skill level, preference and institutional practice. | Consensus achieved (0 strongly disagree, 0 disagree, 0 neutral, 9 agree, 2 strongly agree) |
| 14. Routine repositioning during surfactant administration may not improve the distribution of surfactant relative to maintaining the infant supine; repositioning may increase the risk of endotracheal tube or catheter mal-positioning. | 14. Routine repositioning during surfactant administration may not improve the distribution of surfactant relative to maintaining the infant supine; repositioning may increase the risk of device mal-positioning. | Consensus achieved (1 strongly disagree, 0 disagree, 1 neutral, 5 agree, 4 strongly agree) |
| 15. Surfactant can be administered as a single bolus or divided aliquots, based on provider preference and clinical considerations. | 15. Surfactant can be administered as a single bolus or divided aliquots, based on provider preference, mode of administration, manufacturer recommendation, and clinical considerations. | Consensus achieved (0 strongly disagree, 0 disagree, 1 neutral, 7 agree, 3 strongly agree) |
| 16. After administering surfactant via INSURE technique, infants should be extubated as soon as clinically appropriate. | 16. When using the INSURE technique to administer surfactant, infants should be extubated as soon as possible. | Consensus achieved (0 strongly disagree, 0 disagree, 1 neutral, 7 agree, 3 strongly agree) |
| 17. For spontaneously breathing infants on continuous positive airway pressure (CPAP) with RDS, less-invasive methods of surfactant administration may be appropriate alternatives to the INSURE technique. | 17. For spontaneously breathing infants on continuous positive airway pressure (CPAP) with RDS for whom the decision to give surfactant has been made, less-invasive methods of surfactant administration may be appropriate alternatives to the INSURE technique. | Consensus achieved (1 strongly disagree, 0 disagree, 0 neutral, 5 agree, 5 strongly agree) |
| 18. For preterm infants with RDS who are spontaneously breathing with adequate respiratory effort, LISA and MIST may be appropriate less-invasive options for surfactant administration based on provider experience and institutional practice. | 18. For preterm infants with RDS with adequate respiratory effort on CPAP (not requiring invasive mechanical ventilation), LISA/MIST are appropriate less-invasive options for surfactant administration based on provider experience and institutional practice. | Consensus achieved (0 strongly disagree, 1 disagree, 1 neutral, 4 agree, 5 strongly agree) |
| 19. Surfactant administration via LMA could benefit certain populations of preterm infants, especially when provider experience with other methods of administration is limited. This is an area that needs further research. | 19. Surfactant administration via supraglottic airway devices (eg, LMA) may benefit certain populations of preterm infants and is a promising method of surfactant administration. | Consensus achieved (1 strongly disagree, 1 disagree, 0 neutral, 5 agree, 4 strongly agree) |
| 20. Not enough evidence exists to support the routine use of aerosol administration of surfactant. | 20. More data are required to evaluate the use of the promising technique of aerosol administration of surfactant. | Consensus achieved (0 strongly disagree, 0 disagree, 1 neutral, 7 agree, 3 strongly agree) |
| 21. More research in and more experience with novel methods of surfactant administration (e.g., LMA, aerosol) should be conducted, especially in smaller infants. | 21. More research in and more experience with novel methods of surfactant administration (eg, LMA, aerosol) is needed in smaller, less-mature infants. | Consensus achieved (1 strongly disagree, 0 disagree, 0 neutral, 6 agree, 4 strongly agree) |
| **Section 3 (Workshop 2)** | | |
| 22. Pain management, physical discomfort, and procedural success are all considerations around premedication usage in surfactant administration. | 22. Premedication usage for surfactant administration depends on the method of administration. Pain management, physical discomfort, procedural success, and minimization of adverse events are all considerations around premedication usage in surfactant administration. | Consensus achieved (0 strongly disagree, 0 disagree, 0 neutral, 6 agree, 5 strongly agree) |
| 23. More evidence is required to recommend routine sucrose use before administration of surfactant. | 23. More studies are needed to evaluate the safety and efficacy of sucrose use as a pre-medication prior to administration of surfactant. | Consensus achieved (0 strongly disagree, 2 disagree, 0 neutral, 7 agree, 2 strongly agree) |
| 24. IV atropine is not routinely used for surfactant administration, however, in certain circumstances, it can be used to reduce incidence of bradycardia occurring secondary to vagal stimulation. | 24. Intravenous (IV) atropine is not routinely used for less-invasive methods of surfactant administration. | Consensus achieved (0 strongly disagree, 2 disagree, 0 neutral, 6 agree, 3 strongly agree) |
| 178. In preterm infants with a diagnosis of RDS, when the clinical decision has been made to administer surfactant, giving surfactant early (≤2 hours of life) improves outcomes and is appropriate for any preterm infant with RDS who meets the dosing criteria including preterm infants intubated due to RDS. | 178. When the clinical decision has been made to administer surfactant, preterm infants with RDS should receive surfactant early (≤2 hours of life), preferably within 1 hour of life. | Consensus achieved (0 strongly disagree, 2 disagree, 0 neutral, 9 agree, 0 strongly agree) |
| 2910. For preterm infants with RDS, an elevated FiO2 (≥ 0.3) while on respiratory support is an important indicator for the need of early surfactant treatment (within 2 hours of life) for RDS based on dosing criteria; other clinical measures of respiratory distress and/or support, alone or in combination, may precede and preempt FiO2 as indication of need for surfactant treatment. | 2910. For pre-term infants with RDS receiving positive pressure support, an elevated and increasing FiO_2_ is an important indicator of the need for surfactant treatment based on administration criteria; other clinical measures of respiratory distress and/or support, alone or in combination, may precede and preempt FiO_2_ as indication of need for surfactant treatment. | Consensus achieved (0 strongly disagree, 0 disagree, 0 neutral, 8 agree, 3 strongly agree) |
| 11. Lung ultrasound and clinical respiratory scoring (e.g., RSS) may be useful as an adjunct tool to determine the need for surfactant treatment for non-intubated infants with RDS. | 11. Additional studies are needed to assess the role of lung ultrasound and clinical respiratory scoring as adjunct tools in determining the need for surfactant administration for nonintubated infants with RDS. | Consensus achieved (0 strongly disagree, 0 disagree, 0 neutral, 8 agree, 3 strongly agree) |

1. Evaluation of Respiratory Distress Using Downes’ Score

|  | 0 | 1 | 2 |
| --- | --- | --- | --- |
| Respiratory Rate | <60/min | 60-80/min | >80/min |
| Retractions | No retraction | Mild retractions | Severe retractions |
| Cyanosis | No cyanosis | Cyanosis relieved by O_2_ | Cyanosis on O_2_ |
| Air Entry | Good bilateral air entry | Mild decrease in air entry | No air entry |
| Grunting | No grunting | Audible by stethoscope | Audible with ear |

Scoring - Score <4: no/mild respiratory distress. Score 4 to 7: respiratory distress. Score >7: impending respiratory failure. Adapted from Vohra R, Singh V, Bansal M, Pathak D. Respiratory and Gastrointestinal Involvement in Birth Asphyxia. *Academic Journal of Pediatrics & Neonatology*. 2018;6(4).
